# Rebalancing Hemostasis: Fitusiran as a First‐in‐Class RNAi Therapy in Hemophilia A and B

**DOI:** 10.1002/hsr2.71702

**Published:** 2025-12-29

**Authors:** Raza Ur Rehman, Rida Fatima, Aymar Akilimali

**Affiliations:** ^1^ Shaikh Khalifa Bin Zayed Al‐Nahyan Medical and Dental College Lahore Pakistan; ^2^ Department of Research Medical Research Circle (MedReC) Goma Democratic Republic of the Congo

## Abstract

**Background and Aims:**

Fitusiran is a first‐in‐class RNA interference (RNAi) therapy that lowers antithrombin (AT) to rebalance hemostasis in people with Hemophilia A or B, with or without inhibitors. Its recent FDA approval marks a major shift toward factor‐independent prophylaxis. This commentary aims to summarize the clinical evidence supporting fitusiran, highlight its therapeutic significance, and discuss its potential advantages and safety considerations.

**Methods:**

This commentary reviews key findings from Phase 1–3 clinical trials, including ATLAS‐INH and ATLAS‐PPX, as well as regulatory documents and published pharmacologic evaluations. Evidence on annualized bleeding rate (ABR) reduction, thrombin generation, safety outcomes, and patient‐reported benefits was synthesized to assess the impact of AT suppression on bleed prevention.

**Results:**

Across clinical development, fitusiran produced substantial reductions in bleeding rates in Hemophilia A and B, irrespective of inhibitor status. Phase 1 and 2 trials demonstrated > 70%–80% AT suppression with marked increases in thrombin generation. In Phase 3 trials, median ABR decreased from 17.7 to 0.0 in ATLAS‐INH and by approximately 65% in ATLAS‐PPX after switching from standard prophylaxis. Many participants remained completely bleed‐free. Safety findings included reversible elevations in liver enzymes, mild injection‐site reactions, headaches, and rare thrombotic events, necessitating AT‐level and hepatic monitoring.

**Conclusion:**

Fitusiran offers a transformative, once‐monthly prophylactic option with broad applicability across hemophilia types and inhibitor status. Its RNAi‐based, factor‐independent mechanism enables durable bleed protection and improved treatment convenience. While long‐term hepatic safety and thrombotic risk require continued surveillance, fitusiran represents a significant advance in hemophilia management and a promising step toward more accessible, individualized, and rebalancing‐based therapies.

## 1 Introduction

On March 28, 2025, the U.S. FDA approved Qfitlia (fitusiran) for routine prophylaxis to prevent or reduce bleeding in adults and adolescents ≥ 12 years with Hemophilia A or B, with or without inhibitors. Qfitlia is the first antithrombin (AT)‐directed small interfering RNA (siRNA) therapy that rebalances hemostasis by reducing hepatic AT synthesis. The FDA‐approved dosing follows an AT‐based dose regimen (AT‐DR) titrated to maintain AT activity 15%–35%, with AT‐level monitoring using an FDA‐cleared assay. AT‐based dose regimen (titrated to maintain 15%–35% AT activity) regimen used in clinical trials is not approved in the current U.S. labeling due to higher thrombotic and gallbladder risks. It represents the first approved RNA interference (RNAi)‐based agent for hemophilia, marking a major therapeutic advancement by offering a novel, factor‐independent mechanism and subcutaneous dosing, transforming the prophylactic landscape for both inhibitor and noninhibitor patients [1].

### 1.1 Hemophilia A and B

Hemophilia A and B are congenital X‐linked bleeding disorders caused by a deficiency of Factors VIII and IX, respectively. Hemophilia A occurs in approximately 1 in 5000 male births, while Hemophilia B occurs in about 1 in 25,000 male births. Clinical severity is categorized as mild, moderate, or severe based on baseline factor activity [2]. The prevalence of hemophilia is higher in developed countries due to improved survival and diagnostics. Data suggests that approximately 400,000 people globally live with hemophilia, but only 25%–30% are properly diagnosed. Treatment disparities remain a significant global challenge [3]. The majority of patients with hemophilia live in developing countries where access to factor therapy is limited. About 75% of people with hemophilia globally receive little or no treatment, contributing to a higher burden of joint disease, disability, and early death [4]. One of the most serious complications in hemophilia treatment is the development of inhibitors to infused clotting factors, particularly in severe Hemophilia A, occurring in up to 30% of patients. These inhibitors neutralize the therapeutic efficacy of factor concentrates [5]. Hemophilia often presents in the first year of life with spontaneous bleeds, especially into joints and soft tissues. Early diagnosis is critical, and prophylactic treatment started in childhood can prevent most complications, including disabling hemarthrosis and joint deformities [6].

### 1.2 Current Treatment Options

Current treatment options for Hemophilia A and B include factor replacement therapy, extended half‐life products, nonfactor coagulation products like subcutaneous emicizumab, rebalancing hemostatic agents, and emerging gene therapy approaches under late‐phase clinical investigation. Factor VIII (for Hemophilia A) and Factor IX (for Hemophilia B) replacement therapies remain the gold standard for managing bleeding episodes [7]. Treatment options include factor replacement therapy with plasma‐derived or recombinant Factor VIII/IX concentrates, used as on‐demand or prophylaxis. Prophylaxis is the gold standard in severe hemophilia, aiming to prevent bleeds and joint disease [2]. Extended half‐life (EHL) factor products, developed through Fc fusion or PEGylation, allow for less frequent dosing and improved adherence. These agents maintain factor levels above the bleeding threshold for longer durations, enhancing prophylactic efficacy [8]. Emicizumab, a bispecific monoclonal antibody, mimics Factor VIII function and is approved for prophylaxis in Hemophilia A with or without inhibitors. It offers subcutaneous administration and has significantly reduced bleeding rates in clinical trials [9]. Bypassing agents like recombinant activated Factor VII and prothrombin complex concentrates are used for patients with high‐titer inhibitors [10]. Recent advancements have led to gene therapies that can normalize factor levels, though they come with variability in efficacy and potential long‐term risks. Research is ongoing into nonviral gene transfer systems and gene editing techniques to enhance treatment efficacy and safety [11]. Despite advancements in factor replacement and nonfactor therapies, many patients, especially those with inhibitors, still experience breakthrough bleeds, treatment burden, and poor adherence due to frequent infusions. QFITLIA (fitusiran) fills this gap by offering a once‐monthly subcutaneous RNAi therapy that rebalances coagulation by targeting AT, independent of Factor VIII or IX. It is the first RNAi‐based hemophilia drug, effective across Types A and B, with or without inhibitors. Its approval addresses the unmet need for a universal, durable, and convenient prophylactic option in hemophilia care.

### 1.3 Comparison With Other Nonfactor Therapies

Emicizumab, a bispecific monoclonal antibody, mimics Factor VIII cofactor activity and is approved only for Hemophilia A. Fitusiran, by contrast, lowers AT to rebalance coagulation in both A and B types, regardless of inhibitor status. Although both agents are subcutaneous and reduce bleeding substantially, fitusiran offers broader applicability while requiring routine AT‐level monitoring to mitigate thrombosis risk [12].

### 1.4 Comparison With Gene Therapy Approaches

AAV‐based gene‐transfer products such as valoctocogene roxaparvovec and etranacogene dezaparvovec restore Factor VIII or IX expression for several years but show variable durability, immune responses, and transaminitis. Fitusiran achieves reversible, titratable rebalancing of hemostasis without viral vector integration, making it suitable even for patients with prior inhibitor development or hepatic contraindications [13].

### 1.5 Targeting AT: Fitusiran's Mechanism

**Figure 1:**
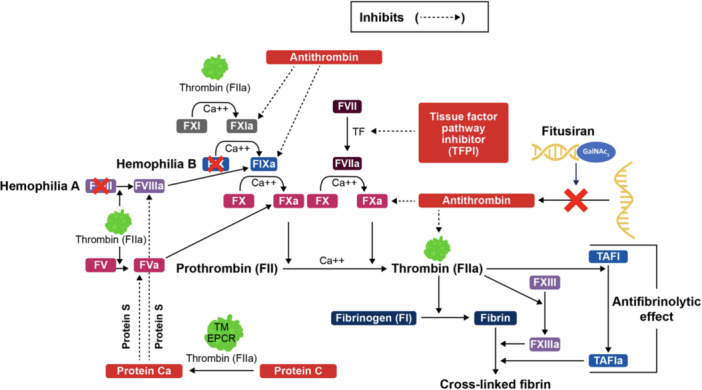
Mechanism of action of fitusiran in rebalancing hemostasis. The schematic shows how RNA interference‐mediated suppression of hepatic antithrombin synthesis increases thrombin generation and restores hemostatic balance in Hemophilia A and B. EPCR, endothelial Protein C receptor; FII, prothrombin; FIIa, thrombin; FV, FVII, FIX, and FX, coagulation factors.

Fitusiran is a synthetic siRNA therapeutic that targets AT mRNA in hepatocytes. By targeting the liver, fitusiran binds to and degrades the messenger RNA of AT, effectively silencing its gene expression and reducing AT synthesis. This increases thrombin generation, thereby restoring hemostatic balance in patients with Hemophilia A or B, regardless of inhibitor status [14, 15]. This mechanism leads to a dose‐dependent decrease in AT levels, which has been shown to improve clinical outcomes, such as reducing annualized bleeding rates (ABRs) and enhancing perioperative hemostasis [16, 17]. Figure 1 [18] illustrates the coagulation cascade in hemophilia, highlighting the inhibitory role of AT and the therapeutic action of fitusiran. By reducing AT levels, fitusiran restores thrombin generation and promotes effective hemostasis.

### 1.6 Objective

This commentary discusses fitusiran's clinical development and therapeutic significance as a first‐in‐class RNAi therapy. By highlighting outcomes from Phase 1–3 studies, it explores how AT suppression rebalances hemostasis and addresses challenges such as inhibitor formation and treatment burden. We summarize key findings from recent Phase 1, 2, and 3 trials, discussing its efficacy in reducing bleeding rates and its novel RNAi‐based, factor‐independent mechanism. By connecting molecular innovation with clinical outcomes, this article highlights the significance of fitusiran's FDA approval and its role in addressing unmet needs in the prophylactic management of hemophilia.

## 2 Discussion

### 2.1 Clinical Evidence and Therapeutic Perspective

#### 2.1.1 Phase 1 Clinical Trial

**Figure 2:**
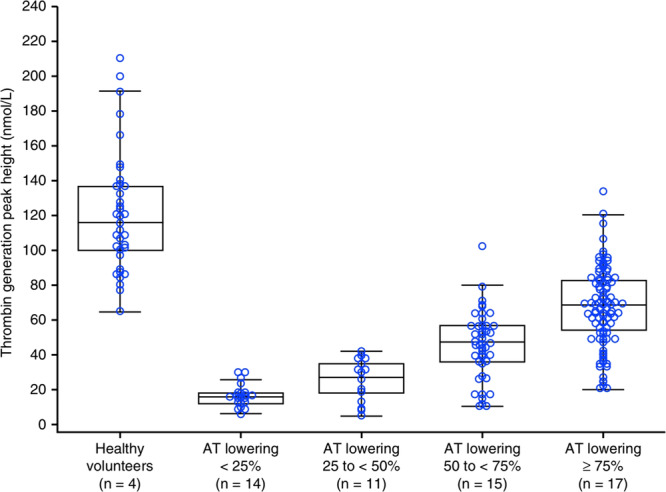
Post hoc analysis of thrombin generation associated with antithrombin (AT) reduction in patients with Hemophilia A or B with inhibitors. For all patients, at each time point, an AT level and a corresponding thrombin generation measurement were recorded. All available thrombin generation values were associated with an AT activity level relative to baseline and were binned into AT‐lowering quartiles. Boxes denote median and interquartile ranges. The middle line in the boxes denotes median values, and top and bottom of the boxes represent the interquartile ranges. Minimum and maximum values are shown by the bars (excluding outliers). Healthy volunteer data were previously presented in Pasi et al.^19^ *Note:* AT inhibits coagulation Factor VII, and its reduction enhances thrombin generation.

From early‐phase exploration to pivotal trials, the clinical development of fitusiran has provided evidence supporting its efficacy and safety profile. The study involved 25 male participants, including 16 with Hemophilia A or B (without inhibitors) and 9 healthy volunteers, to evaluate the effects of subcutaneous fitusiran administered in both single and multiple ascending doses. The results showed that fitusiran led to a mean reduction of 70%–80% in AT levels, which was accompanied by increased thrombin generation in all patients. Bleeding episodes were reduced, and the therapeutic effect lasted for several weeks after the dose. The safety profile was deemed acceptable, with no serious adverse events reported during Phase 1. These findings provided proof‐of‐concept for RNAi targeting AT to restore coagulation and marked the initiation of a novel class of nonfactor therapies for hemophilia. Post hoc analysis of thrombin generation associated with AT reduction in patients with Hemophilia A or B with inhibitors is shown in Figure 2 [19].

Preclinical and early Phase 1 pharmacologic modeling provided critical support for the dosing rationale aimed at AT suppression in humans. The study demonstrated clear dose‐response relationships between siRNA exposure, AT reduction, and thrombin restoration. Additionally, it provided evidence of a long half‐life and sustained AT knockdown following monthly dosing. These insights were essential in defining the optimal dose regimens and enabled the successful transition into Phase 1 human studies [20].

#### 2.1.2 Phase 2 Clinical Trial

Phase 2 dose‐escalation study evaluated escalating doses of fitusiran (ranging from 50 to 80 mg monthly) to assess dose–response effects in patients with Hemophilia A or B without inhibitors. AT levels were effectively suppressed by > 80%, leading to increased thrombin generation. Bleeding episodes significantly decreased, and several participants reported no bleeds at all. This trial laid the groundwork for determining the optimal prophylactic dosing for Phase 3 trials [21]. Fitusiran Phase 2 study in hemophilia with inhibitors focused on patients with inhibitors to FVIII or FIX. This trial assessed monthly fitusiran injections. Participants experienced significantly reduced ABRs, and nearly half had no treated bleeds during the 6‐month evaluation. Improvements in quality of life and physical activity were also reported. The trial supported fitusiran as a potential universal prophylactic across inhibitor status. Mild liver enzyme elevations were the main safety concern, managed with regular monitoring [22].

#### 2.1.3 Phase 3 Clinical Trial

**Figure 3:**
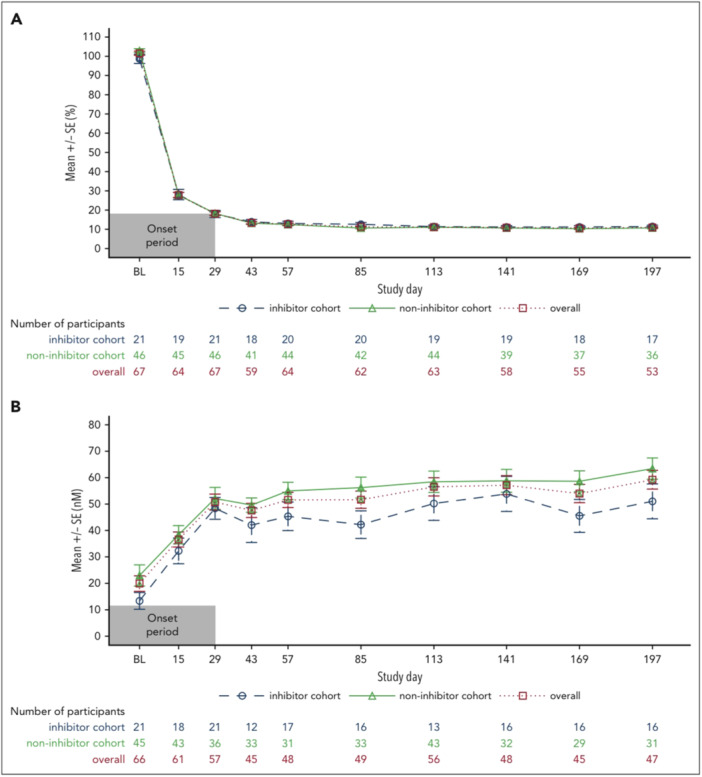
Pharmacodynamic outcomes. (A) Mean change in AT levels from baseline. (B) Mean peak TG. Measurements after fitusiran discontinuation +28 days were excluded. Measurements from the start date of heparin, AT concentrate, and FXa inhibitor to the final date on which those products were administered, plus five half‐lives of that specific product, were excluded. Measurements in the period of missing at least two consecutive fitusiran doses were excluded. Only central laboratory assessments were taken into account. SE, standard error.

The ATLAS‐INH Phase 3 trial evaluated fitusiran in patients with Hemophilia A or B and inhibitors, comparing it to on‐demand treatment with bypassing agents (aPCC or rFVIIa). The study showed a dramatic reduction in median ABR from 17.7 in the control group to 0.0 in the fitusiran arm, demonstrating its superior bleed prevention. Analytically, this result is clinically significant—patients with inhibitors often suffer frequent bleeds and poor quality of life due to limited prophylactic options. Fitusiran's mechanism, targeting AT via RNAi, restores thrombin generation and offers consistent protection, regardless of inhibitor status. The once‐monthly subcutaneous administration also enhances treatment adherence. However, the study noted ALT elevations in some participants, highlighting the need for ongoing hepatic monitoring. Overall, ATLAS‐INH establishes fitusiran as a transformative therapy for a historically underserved patient group [23]. The ATLAS‐PPX study was a pivotal Phase 3 trial aimed at assessing the clinical benefit of switching patients with Hemophilia A or B—with or without inhibitors—from their existing standard prophylaxis regimens (either factor replacement or bypassing agents) to fitusiran prophylaxis. This crossover design allowed for direct comparison of bleed control within the same individuals before and after the transition. Clinically, the trial demonstrated a 65% reduction in ABR after switching to fitusiran, highlighting a substantial improvement in bleed protection. The magnitude of this reduction is especially important given the study's diverse patient pool, which included both inhibitor and noninhibitor populations—underscoring fitusiran's broad applicability and consistent efficacy. Additionally, patient‐reported outcomes showed significant improvements in quality of life, particularly in physical activity, emotional well‐being, and treatment satisfaction. Pharmacodynamic outcomes are shown in Figure 3 [24]. Table 1 summarizes the pivotal Phase III trials (ATLAS‐INH and ATLAS‐PPX), highlighting design, efficacy, and safety outcomes.

**Table 1:** Summary of Phase III fitusiran trials (ATLAS‐INH and ATLAS‐PPX).

Parameter	ATLAS‐INH	ATLAS‐PPX
Population	Hemophilia A/B with inhibitors	Hemophilia A/B ± inhibitors
Design	Phase III, randomized, open‐label	Phase III, crossover
Intervention	Fitusiran 80 mg SC monthly (trial dose)	Fitusiran 80 mg SC monthly (trial dose)
Comparator	On‐demand bypassing agents (aPCC, rFVIIa)	Prior factor or BPA prophylaxis
Median ABR	0.0 versus 17.7	1.5 versus 4.3
ABR reduction	≈100%	≈65%
Bleed‐free patients	50%	43%
Main AEs	↑ALT/AST (15–20%), injection‐site rxn (< 10%)	Mild transaminase ↑, injection‐site rxn (< 10%)
Serious AEs	One thrombotic event (early study)	None fatal
Monitoring	AT 15%–35%, liver tests q1–3 mo	Same protocol

### 2.2 Safety and Tolerability Profile

#### 2.2.1 Most Common Adverse Effects

**• Elevated Liver Enzymes (ALT/AST Elevations)**

Mild to moderate transaminase elevations were observed in 15%–20% of patients, often asymptomatic but requiring monitoring or temporary dose interruption [25].

• **Injection Site Reactions**

Mild erythema and discomfort were seen in up to 10% of participants, but none led to treatment discontinuation [26].

• **Headaches and Fatigue**

Reported in 8%–12% of patients, these side effects were mild and self‐resolving without medical intervention [27].

• **Thrombotic Events (Rare)**

One fatal thrombotic event in early trials led to cautious dose revision in future studies and tighter eligibility criteria.

• **Mild Anemia**

Mild decreases in hemoglobin were noted (~6%), requiring regular monitoring but no treatment discontinuations [28].

### 2.3 Long‐Term Monitoring and Dose Adjustments

• **Liver Function Monitoring**

ALT/AST monitoring every 1–3 months was recommended, with dose interruptions for significant elevations [29].

• **Thrombotic Risk Surveillance**

Enhanced thrombotic risk monitoring was introduced post‐fatality, including exclusion of high‐risk individuals [30].

• **Patient Symptom Education**

Patients were educated on early symptoms of thrombotic events and advised prompt reporting to reduce risks [31].

• **Thrombotic‐Risk Mitigation and Monitoring**

Maintain plasma AT activity between 15% and 35%. Monitor AT levels monthly using an FDA‐cleared assay; suspend or reduce dose if AT < 15%. Patients with additional thrombotic risk factors (e.g., obesity, immobility) require closer surveillance and education about early symptoms of thrombosis.

• **Quality of Life Monitoring**

Switching to once‐monthly injections greatly improved patient satisfaction and adherence rates [23].

### 2.4 Final Analysis

Based on cumulative clinical data, fitusiran yielded a superior efficacy profile with a tolerable safety risk. Abnormal elevations of liver enzymes and occasional thrombotic adverse effects need to be monitored, although newer regimen schemes are improving overall tolerance. Fitusiran is a potential universalized prophylaxis for patients with Hemophilia A or B [32]. Larger real‐world studies and longer follow‐up will be crucial to determining its place within clinical practice.

### 2.5 Fitusiran's Potential to Redefine Treatment Paradigms in Hemophilia

Fitusiran brings a new paradigm to hemophilia treatment through targeting a physiological anticoagulant, AT, instead of replacing deficient coagulating factors, a new rebalancing approach [33]. This is promising for both patients with inhibitors as well as for those who have been treated historically with factor replacement therapies. Contrasting with multiple weekly doses of prophylactic factor infusions, monthly subcutaneous dosing with fitusiran facilitates patient compliance and lifestyle flexibility [34]. Significant bleeding episode reduction through various patient subgroups in recent trials (ATLAS‐PPX, ATLAS‐INH) confirmed its wide range of application [23]. Furthermore, this mechanism also promises therapies for other rare bleeding diseases outside Hemophilia A and B, opening new therapeutic horizons. Future clinical pathways may increasingly focus on rebalancing agents like fitusiran, reshaping hemophilia care toward disease modulation rather than replacement [31].

### 2.6 Addressing the Burden of Inhibitor Development

One of the most severe hurdles to hemophilia management is the formation of inhibitors, which makes it more difficult to treat and more expensive. Fitusiran's approach—AT targeting instead of missing factor replacement—provides a new way to circumvent the problem of inhibitors entirely [35]. This represents a fundamental shift in how physicians may approach prophylaxis and long‐term disease control in this difficult subgroup.

### 2.7 Economic and Healthcare Benefit of Fitusiran

In pivotal Phase 3 trials, median ABR decreased from 17.7 to 0.0 in ATLAS‐INH and from 4.3 to 1.5 in ATLAS‐PPX—representing approximately 100% and 65% reductions, respectively, compared with on‐demand or prior prophylaxis regimens. These results reflect the fixed‐dose (80 mg monthly) trial design and not the current FDA‐approved AT‐based dose regimen (15%–35% AT activity) used in clinical practice [31]. The reduction in bleeding events may contribute to fewer emergency visits and hospitalizations; however, these potential savings remain theoretical. Early modeling studies suggest possible cost offsets through decreased use of bypassing agents, but definitive cost‐effectiveness or real‐world utilization data are not yet available [35].

### 2.8 Boxed Warning (U.S. Label)

Qfitlia (fitusiran) increases the risk of thrombosis and gallbladder disease. Reported events include cerebral venous thrombosis, deep‐vein thrombosis, and cholecystitis. Therapy must be interrupted if AT activity < 15% or if clinical signs of thrombosis occur. Patients should be educated to promptly report abdominal pain or neurologic symptoms.

### 2.9 Bleed‐Management Precautions

During breakthrough bleeds, limit the use and intensity of bypassing agents or factor concentrates to the lowest effective dose to avoid excessive thrombin generation.

### 2.10 Hepatic Impairment

Qfitlia is not recommended in moderate‐to‐severe hepatic impairment because of altered AT synthesis and increased risk of adverse reactions.

## 3 Conclusion

Fitusiran represents a paradigm shift in the prophylactic management of Hemophilia A and B, offering a novel, factor‐independent mechanism that rebalances coagulation through targeted AT suppression. Clinical trials across all phases have demonstrated substantial reductions in bleeding rates (≈65%–100%) under fixed‐dose trial conditions, while ongoing real‐world use follows individualized AT‐based dosing for optimized safety. However, the risks of liver enzyme elevations and rare thrombotic events necessitate vigilant monitoring and individualized dosing strategies. Economic barriers, including high upfront costs, highlight the importance of health policy reforms to enable equitable access. Ongoing real‐world studies and long‐term safety trials will be pivotal in consolidating its role within clinical practice. As RNAi therapeutics advance, fitusiran stands poised to redefine hemophilia care by offering a durable, simplified, and transformative prophylactic option for a historically underserved patient population.

## Author Contributions

**Raza Ur Rehman:** conceptualization, investigation, methodology, visualization, writing – review and editing, writing – original draft, formal analysis, software, data curation, validation, supervision. **Rida Fatima:** writing, review, supervision and validation. **Aymar Akilimali:** funding acquisition, project administration, resources, supervision, writing – review and editing.

## Funding

The authors received no specific funding for this work.

## Conflicts of Interest

The authors declare no conflicts of interest.

## Transparency Statement

The lead author Aymar Akilimali affirms that this manuscript is an honest, accurate, and transparent account of the study being reported; that no important aspects of the study have been omitted; and that any discrepancies from the study as planned (and, if relevant, registered) have been explained.

## Data Availability

Data sharing is not applicable to this article as no datasets were generated or analyzed during the current study.
